# Influence of Neighborhood Factors on Sleep in Home Dwelling Multiethnic Older Adults With Dementia

**DOI:** 10.1093/geroni/igaa057.2105

**Published:** 2020-12-16

**Authors:** Adriana Perez, Augestine Boateng, Sonia Talwar, Nancy Hodgson

**Affiliations:** 1 University of Pennsylvania, Philadelphia, Pennsylvania, United States; 2 University of Pennsylvania, Cherry Hill, New Jersey, United States

## Abstract

Current scientific paradigms inadequately capture complex clinical, behavioral, and sociocultural factors impacting health and well-being in persons living with dementia (PLWD). The purpose of this study was to identify differences in individual and neighborhood-level factors contributing to sleep among multi-ethnic PLWD. Wrist actigraphy measured objective sleep characteristics. Subjective sleep was assessed using the PROMIS sleep measure. GIS mapping analyzed neighborhood-level factors (walkability, green space, crime index, density). Walkability was significantly associated with subjective sleep (p.006) controlling for age and dementia stage. Number of night awakenings was significantly associated with density, crime and housing value (p<.001). PLWD in neighborhoods with higher population density, annual crime, low median home and low walkability would benefit from interventions targeting unsupportive neighborhood environments to improve sleep.

